# Supplementary material for: External Validation with Accuracy Confounders of VCO2-Derived Predicted Energy Expenditure Compared to Resting Energy Expenditure Measured by Indirect Calorimetry in Mechanically Ventilated Children
Source: Nutrients. 2022 Oct 10;14(19):4211. doi: 10.3390/nu14194211 (PMC9571636; doi:10.3390/nu14194211)
Supplement: Supplementary file 1 [file nutrients-14-04211-s001.zip › nutrients-1929042-supplementary.pdf]

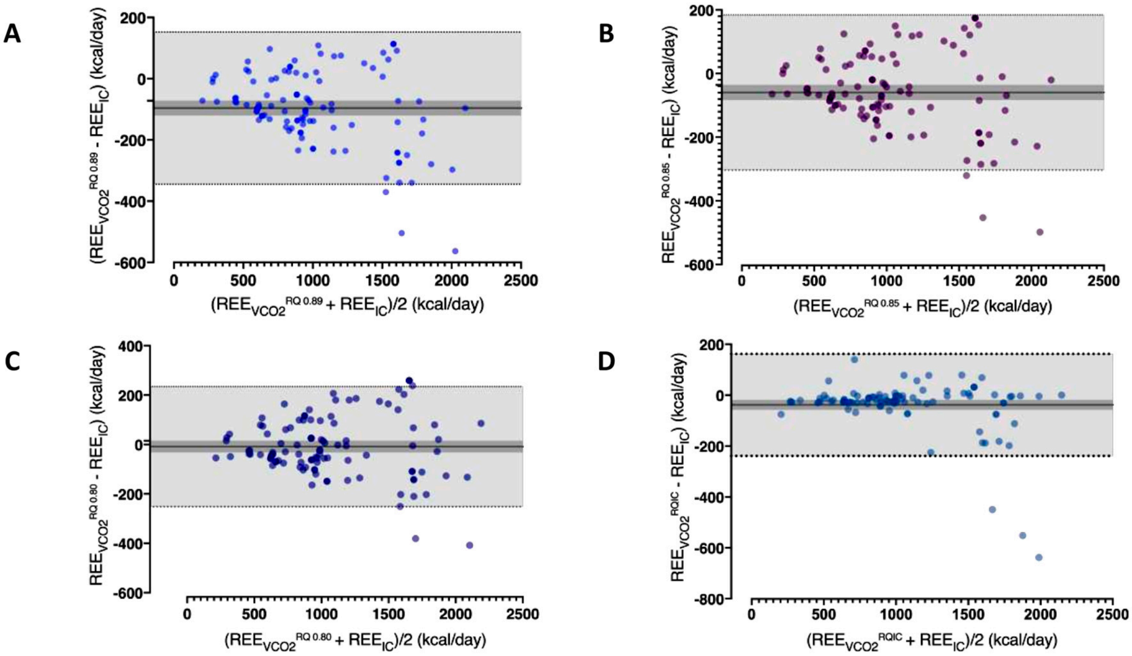

Figure S1: Bland–Altman plots whereby resting energy expenditure (REE) based on volumetric carbon dioxide production (VCO_2) measurements (REE_{VCO2}) using different RQ values are compared to REE measured by indirect calorimetry (IC) (REE_{IC}) at ICU Day-1. Solid lines indicate the bias (kcal/day), and the light shades with the fine dotted lines indicate the limits of agreement (bias ± (1.96 × SD) = precision). Dark shades represent the 95% confidence intervals of the mean (bias). (**A**) $REE_{VCO2}{}^{RQ\ 0.89}$, assuming RQ of 0.89 compared to REE_{IC}. (**B**) $REE_{VCO2}{}^{RQ\ 0.85}$, assuming RQ of 0.85 compared to REE_{IC}. (**C**) $REE_{VCO2}{}^{RQ\ 0.80}$, assuming RQ of 0.80 compared to REE_{IC}. (**D**) $REE_{VCO2}{}^{RQ\ IC}$ using individual RQ values calculated by IC compared to REE_{IC}.
